# Linking Colleague Support to Employees’ Promotive Voice: A Moderated Mediation Model

**DOI:** 10.1371/journal.pone.0132123

**Published:** 2015-07-06

**Authors:** Xiao-Yun Xie, Chu-Ding Ling, Shen-Jiang Mo, Kun Luan

**Affiliations:** 1 School of Management, Zhejiang University, Hangzhou, China; 2 Lingnan University College, Sun Yat-sen University, Guangzhou, China; University of Groningen, NETHERLANDS

## Abstract

Promotive voice is essential for improving team and organization performance. Yet in the current literature, less was known regarding the psychological reasons why people engage in promotive voice. Through the lens of social exchange, we proposed that employees who received support from colleagues may develop higher level of felt obligation for constructive change which leads to promotive voice. Analyses of multi-source data from 51 cross-functional sources (51 team supervisors and 162 employees) showed that employees’ felt obligation for constructive change positively mediates the relationship between colleague support and promotive voice behavior. Moreover, the impact of colleague support on felt obligation for constructive change is stronger when there is a low level of subgroup formation in the team. Theoretical and practical implications of these findings are discussed.

## Introduction

Defined as “*employees’ expression of new ideas or suggestions for improving the overall functioning of their work unit or organization*” [1], promotive voice is beneficial for work teams to facilitate team learning (e.g., [2]), create opportunities for improvement (e.g., [3]), bring about change processes (e.g., [4]), and improve work performance (e.g., [5, 6]). However, as indicated in previous literature, employees generally lack the proactivity to speak up (e.g., [7]). Therefore, it is critical for us to develop better understanding regarding the factors that may foster or suppress promotive voice in work teams.

Past voice literature made significant progress in identifying antecedents of employee voice, such as personal causes (e.g., [8]), and contextual factors (e.g., [9, 10]). Most of them indicated that perceived psychological safety plays as a key mechanism through which employees are encouraged to speak up. However, such a process is less powerful in predicting promotive voice, which actually requires employees’ strong commitment and responsibilities towards the team besides psychological safety [1]. Thus, Liang and colleagues [1] identified felt obligation for constructive change as a unique factor which may significantly enhance employees’ promotive voice. Nevertheless, less is known about factors that may stimulate employees’ felt obligation for constructive change.

The nature of teamwork (e.g., task interdependence) indicates that peer colleagues may have salient impact on individuals’ work attitude and behavior [11, 12]. Drawing on social exchange theory, we propose that support from colleagues may significantly promotes employees’ felt obligation for constructive changes, which in turn motivates promotive voice behaviors in team settings. Additionally, subgroup formation is a pervasive phenomenon which has been recently recognized as an essential factor in shaping employee behaviors (e.g., [13]). Given that employees’ feeling of obligation toward the team may substantively change once subgroups emerge [14, 15], we further propose the moderating role of team level subgroup formation in the relationship between colleague support and felt obligation for constructive changes.

The present study makes several contributions. First, we advance knowledge of antecedents of promotive voice by highlighting the role of felt obligation for constructive changes in enhancing employees’ promotive voice behavior. Second, we adopt a new perspective on exploring antecedents of promotive voice besides previously personal and supervisory factors by focusing on support from peer colleagues in the team settings. Third, we answer prior calls for understanding employee voice from a multilevel perspective [16] by including team level subgroup formation into a cross-level framework. In the following parts of this article, we first review the literature that leads to the establishment of our hypotheses. Subsequently, we test our theory with survey data and explain the major findings. Finally, we discuss the contributions of our study, along with several limitations and directions for future research.

## Theoretical Framework and Hypotheses

### Antecedents of employee promotive voice: Impact from peer colleagues

Past literature indicated that employee voice is conceptualized from various perspectives at different development stages [4, 16, 17]. Employee voice was first defined as a form of employees’ response to dissatisfying conditions in organizations [18–20]. But some researchers argued that employees mainly perceive voice as a form of prosocial behavior, rather than an approach to remove dissatisfaction [16, 17, 21]. Recently, Liang and colleagues [1] significantly extended the voice literature by demonstrating diverse messages expressed through voice and proposing two forms of voice behavior: promotive voice versus prohibitive voice. The ideas incorporated in promotive voice is mainly about innovative suggestions or solutions to improve the status quo, whereas the information provided by prohibitive voice is regarding errors and problematic approaches that are harmful for teams or organizations [1].

Despite both promotive voice and prohibitive voice are constructive and important for team effectiveness, they differ in a meaningful way. Specifically, prohibitive voice mainly reflects employees’ concerns on undesirable practices in work units. Thus it may take more cost associated with personal risk, but less psychological energy to enact [22]. In contrast, promotive voice refers to generating novel suggestions and solutions, which requires sustained cognitive effort and attention. Hence, when considering the inducement of promotive voice, employees’ felt obligation towards their teams is of critical importance [1].

Various antecedents of employee voice have been identified in previous studies. These antecedents include personal causes such as employees’ identification towards their workgroups or organizations (e.g., [8]), and contextual factors such as leader-member exchange (LMX, e.g., [10]) and team climate (e.g., [9]). In spite of the differences in conceptualization, most of these factors are expected to encourage employees to speak up by promoting their perceived psychological safety [16, 17]. However, as discussed earlier, promotive voice is more likely to be expected by employees’ sustained commitment and responsibility, such as felt obligation toward the work unit [1]. Thus, impact mechanisms in current literature on promotive voice may be deficient in explanatory power. Moreover, we have limited knowledge regarding factors that may enhance employees’ felt obligation for constructive change.

The characteristics of shared responsibility and diffuse expertise in teamwork underscore the importance of influences from colleagues (e.g., colleague support) in shaping individuals’ attitude and behavior [11, 12, 23]. Generally, colleague support refers to employees’ belief regarding the extent to which they can seek assistance from their coworkers [24, 25]. Previous research addressed that higher perception of support from colleagues, both instrumental (e.g., task-related information) and emotional (e.g., empathy), may lead to more proactive behaviors and higher creative performance [3, 23, 26]. Thus, using social exchange theory as a basis, in the following sections we will illustrate how colleague support motive employees to make promotive voice via stimulating their feelings of obligation toward teams.

### Colleague support and promotive voice: The mediating role of felt obligation for constructive change

Through the lens of social exchange, previous studies found that individuals who receive emotional and instrumental support from peer colleagues are more likely to reciprocally contribute to the focal team and colleagues by making proactive efforts such as providing constructive suggestions and ideas, i.e., promotive voice (e.g., [3, 26, 27]). We further argue that the relationship between colleague support and promotive voice is mediated by felt obligation for constructive change. Employees’ felt obligation for constructive change is defined as “*an individual’s belief that he or she is personally obligated to bring about constructive change*” [28]. In line with social exchange theory, people tend to reciprocally maintain and further develop relationships with others if they are well treated [29]. Accordingly, felt obligation toward the team can be significantly stimulated when employees receive substantial emotional and instrumental support from peer colleagues (e.g., [30–32]). In addition, promotive voice such as expressing novel ideas requires physical and psychological efforts (e.g., [22]). Thus, as mentioned earlier, promotive voice cannot be expected if employees lack constructive responsibilities and obligations toward the focal team and peer colleagues (e.g., [1]). In line with this reasoning, Liang and colleagues [1] found that employees with a high level of felt obligation for constructive changes are more likely to be effectively motivated to engage in promotive voice behavior.

Based on the above reasoning, we propose the following hypothesis:

*Hypothesis 1: Felt obligation for constructive change positively mediates the relationship between colleague support and promotive voice.*

### Colleague support and felt obligation for constructive change: Subgroup formation as a moderator

Recent studies on employee voice emphasized that employee voice is not only driven by individual-level factors, but also influenced by team-level shared perceptions of existing state (e.g., [9, 33]), such as perception of subgroup formation in teams. Defined as the extent to which a work group is psychologically and behaviorally split into different subgroups [15], subgroup formation is commonly observed in work teams. For example, a task force formed to coordinate a company’s R&D and marketing usually fragments into two subgroups consisting of members drawn from each area, respectively [34]. And once the subgroups form, team members incline to hold favoritism towards their in-groups, and hostility towards their out-groups. Consequently, mistrust and conflict may increase [35, 36], while cohesion and social integration may significantly decrease [37, 38]. Such processes would adversely affect the cooperation and coordination in teams [39].

Accordingly, we argue that subgroup formation will have a negative moderating effect on the relationship between colleague support and felt obligation for constructive change by inhibiting employees’ feeling of reciprocity towards teams. When subgroups are formed and perceived within a team, individuals from different subgroups are psychologically and behaviorally disintegrated (e.g., [14, 36]). As a result, employees tend to perceive more support and trust from ingroup members rather than outgroup members [40]. Accordingly, they may feel more responsible for ingroup members rather than the entire team. Consequently, employees will choose other ways to respond to ingroup colleagues who provide support in a reciprocal manner, rather than make promotive voice which will benefit those outgroup colleagues. Therefore, we propose the following hypotheses:

*Hypothesis 2: Subgroup formation negatively moderates the relationship between colleague support and felt obligation for constructive change. Specifically, the relationship between colleague support and felt obligation for constructive changes is stronger when subgroup formation is low than when it is high.*

*Hypothesis 3: The indirect relationship between colleague support and promotive voice via felt obligation for constructive change is moderated by subgroup formation such that the indirect relationship becomes stronger as subgroup formation is low than when it is high.*

**Fig 1:**
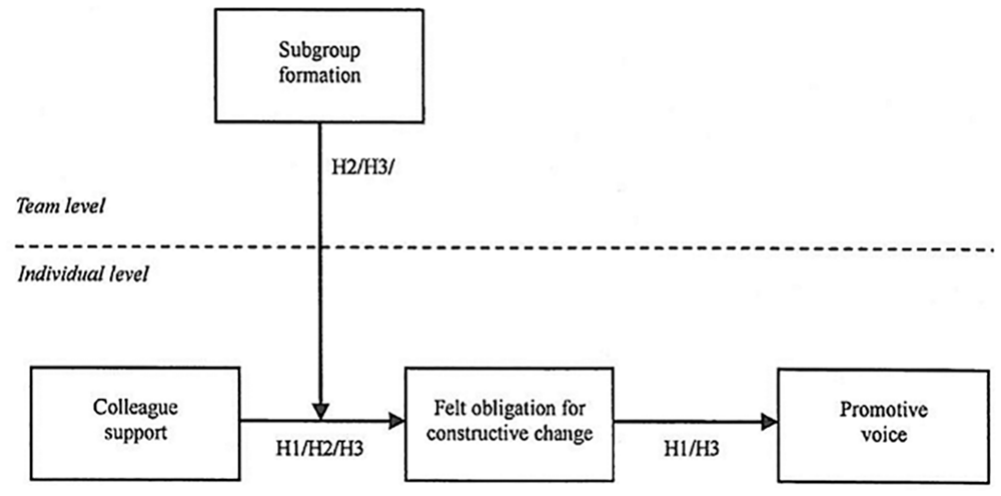
Hypothetical model. H1 represents an indirect effect. H2 represents a cross-level moderation effect. H3 represents a cross-level moderated mediation effect. H = Hypothesis.

All of the hypotheses of this study are shown in Fig 1.

## Methods

### Ethics statement

This study and its consent procedure were approved by the research ethics committee in Institute of Human Resource Management, Zhejiang University. At the very beginning of the study, the employees were verbally informed that all of their individual responses would be used only for academic purposes, and they were asked to complete the questionnaires during work time. The commitment to analyze responses through an anonymous process was explicitly printed on a separate page that was signed by participants and collected afterwards to document this process.

### Sample and procedure

Data in the present study were collected from supervisors and employees working in project teams in IT companies located in East China. It is important for members of these teams to speak up if they intend to change the current situation for themselves, their colleagues and the entire team. Specifically, employees were required to report their demographic information, such as age, gender and tenure, as well as their perception of LMX, colleague support, felt obligation for constructive change, and subgroup formation. Meanwhile, supervisors were kept blind to their employees’ responses. Supervisors completed a questionnaire evaluating the sampled employees’ promotive voice behavior in the teams. All surveys were translated from English to Chinese using Brislin’s [41] recommended translation-back translation procedure.

In total, questionnaires were distributed to 252 employees from 91 teams and their corresponding supervisors. In total, 165 employees from 51 teams returned their questionnaires. Three questionnaires were deleted due to missing data. The final sample comprises 162 employees embedded in 51 teams (with 51 supervisors), and the response rate was 64.3% (data in S1 Data). The average team size was 8.08 members (*SD* = 4.77). Among the 162 employees, 85 were female (52.5%). In terms of age, 0.6% of the participants were under 20 years, 82.7% were between 21 and 30 years, 14.8% were between 31 and 40 years, and the remaining 1.9% were above 40 years. The average team tenure for the respondents was 24.55 months (*SD* = 25.68).

### Measures

Well-established scales were employed to measure the constructs of this study; these are summarized below.

#### Colleague support

Bacharach and colleagues’ [24] four-item peer support scale was used in our survey. The original version was developed based on Caplan and colleagues’ [42] social support scale and was modified to indicate the employee’s perception that he/she receives help from peer colleagues. This scale has also been used in other studies (e.g., [43]). Item examples include “*How much do other team members go out of their way to do things to make your worklife easier for you*?” and “*When things get tough at work*, *how much can you rely on your coworkers for advice or information*”. A five-point Likert response format (1 = *little*; 5 = *very much*) was used. Cronbach’s α was .81.

#### Felt obligation for constructive change

We used Liang and colleagues’ [1] five-item scale to assess the extent to which employees feel an obligation toward fostering team improvement and constructive change. As the original items were designed to measure the extent to which employees felt obligation toward the organization, we modified items by replacing “the organization” with “the team”. An example item for this scale is “*I have an obligation to the team to voice my own opinions*”. Again, a five-point Likert scale format (1 = *strongly disagree* to 5 = *strongly agree*) was applied. The Cronbach’s α of this scale was .91.

#### Subgroup formation

Using Rico and colleagues’ [15] four-item scale, employees were asked to indicate the extent to which they shared a perception that the team was split into different subgroups. This scale has been used in other research (e.g., [44]). An item example is “*In my team*, *internal divisions have arisen during tasks*.” A seven-point Likert scale format was used to assess subgroup formation, ranging from 1 (*strongly disagree*) to 7 (*strongly disagree*). Cronbach’s α was .88.

#### Promotive voice

We assessed employees’ promotive voice using a five-item promotive voice scale developed by Liang and colleagues [1]. Sample items were “*This employee proactively suggests new projects that are beneficial to this team*” and “*This employee proactively voices constructive suggestions that help the team reach its goals*”. Supervisors’ responses were indicated using a seven-point Likert scale (1 = *strongly disagree* to 7 = *strongly agree*). The Cronbach’s α of the promotive voice scale was .93.

#### Control variables

Previous studies indicated that demographic variables, such as age and gender, have a significant impact on employees’ voice behaviors (e.g., [1, 5]); therefore, we controlled for participants’ gender and age in the following data analysis. Employee voice behavior could be influenced by work tenure in the teams [1, 9]. Consequently, respondents’ team tenure was also included as a control variable.

We controlled for the effect of LMX in this research because our study was designed to focus on the impact of colleague support on employees’ promotive voice behaviors and argued that colleague support would explain a significant degree of variance in promotive voice even after we took the leadership factor (leader-member relationship) into account. Therefore, LMX, as a profound antecedent of promotive voice behavior (e.g., [10, 16]), needs to be controlled for. LMX was measured using the seven-item scale developed by Graen and Uhl-Bien [45]. An item example was “*I maintain an effective working relationship with my supervisor*.” A seven-point Likert scale (1 = *strongly disagree* to 7 = *strongly agree*) was used. The Cronbach’s α of the LMX scale was .87. In addition, we also controlled for individual level team identification, team level justice climate and team size because past literature indicated that these variables are influential for employee voice (e.g., [8]). Specifically, team identification was measured by Mael and Ashforth’s [46] 6-item scale using a seven-point Likert scale (1 = *strongly disagree* to 7 = *strongly agree*). An item example was “*When someone criticizes my team*, *it feels like a personal insult*.” Procedural justice climate was assessed with a 7-item scale validated in Colquitt’ work [47]. Again, a seven-point Likert scale (1 = *to a small extent* to 7 = *to a large extent*) was used. An item example was “*To what extent team members were able to express their views and feelings during procedures*.”

### Confirmatory factor analysis

Confirmatory factor analyses were conducted to examine whether employees’ scores on their self-report measures (i.e., colleague support, felt obligation for constructive change, subgroup formation) captured distinctive constructs. The hypothesized three-factor model was specified by loading indicators on their respective latent variables, and the correlations among latent variables were freely estimated. The results showed that the three-factor model fit the data well, *χ* ^2^ (62, *N* = 162) = 96.11, comparative fit index (CFI) = .97, Tucker—Lewis index (TLI) = .96, standardized root-mean-square residual (SRMR) = .05, and root-mean-square error of approximation (RMSEA) = .06. The indicators all significantly loaded on their respective latent factors. Considering that the item contents in some measures were similar to each other, we compared the hypothesized three-factor model with several alternative models. As shown in Table 1, all of the alternative models fit the data significantly worse than the three-factor model. Therefore, the measures reported by employees captured distinct constructs in this study.

**Table 1:** Confirmatory factor analysis of key variables in the study.

Factor structure model	*χ* ^2^ *(df)*	*χ* ^2^ */df*	CFI	TLI	SRMR	RMSEA	*Δχ* ^2^ *(Δdf)*
Three factor (hypothesized): Colleague support, felt obligation, subgroup formation	96.11(62)	1.55	.97	.96	.05	.06	
Two factor							
Model 1 (alternative): Colleague support and felt obligation constrained as one factor	293.07(64)	4.58	.78	.73	.11	.16	196.96(2)
Model 2 (alternative): Felt obligation and subgroup formation constrained as one factor	517.90(64)	8.09	.56	.47	.23	.21	421.79(2)
Model 3 (alternative): Colleague support and subgroup formation constrained as one factor	358.06(64)	5.59	.72	.66	.17	.18	261.95(2)
One factor (alternative): All three scales together as one factor	565.42(65)	8.70	.52	.42	.24	.18	469.31(3)

Note. *N* = 162. All *χ* ^2^ and *Δχ* ^2^ values are significant at *p* < .05. CFI = comparative fit index; TLI = Tucker—Lewis index; SRMR = standardized root-mean-square residual; RMSEA = root-mean-square error of approximation.

### Data aggregation

The interrater agreement index, *r*_wg_ [48], and the intraclass correlation coefficient (ICC) [49] were used as standards for aggregating individual employees’ ratings of subgroup formation into a higher team-level variable. Generally, when the *r*_wg_ is higher than 0.70, the ICC (1) is higher than 0.12, and the ICC (2) is higher than 0.60, an aggregation can result in a more reliable measure of the construct of interest than using a rating from a single individual [50]. In this study, the *r*_wg_ for subgroup formation was 0.83, which demonstrated acceptable within-team agreement on the construct. Meanwhile, the ICC (1) (James, 1982) and ICC (2) [49] for subgroup formation were 0.37 and 0.65, respectively, also providing strong evidence of adequate within-team agreement. In addition, the one-way analysis of variance (ANOVA) results showed that there were significant differences in the team-level means of the subgroup formation ratings, *F*(50, 111) = 2.82, *p* < .01. Taken together, these lines of evidence supported the aggregation of individual level data to the team level.

### Analytic strategies

In the present study, Mplus 6.0 software [51] was used to examine all hypotheses proposed in a multilevel frame. In addition, the Monte Carlo method recommended by Preacher, Zyphur, and Zhang [52] was used to estimate the confidence intervals for the hypothesized mediating effects and multilevel moderating effects to determine their significance. More information about the R program can be found at http://www.quantpsy.org. All standard errors of the model parameter estimates were computed using a sandwich estimator to correct potential sampling bias caused by unequal numbers of participants on each team.

## Results

The means, standard deviations, and bivariate correlations among the studied variables are shown in Table 2. At the individual level (employee level), colleague support is positively correlated with employees’ felt obligation (*r* = 0.45, *p* < .01), while employees’ felt obligation was positively correlated with employees’ promotive voice (*r* = 0.30, *p* < .01). These findings provided preliminary support for the hypothesized relationships.

**Table 2:** Means, standard deviations, and bivariate correlations among studied variables.

Variables	*M*	Ind. *SD*	Team *SD*	1	2	3	4	5	6	7	8	9	10	11
*Individual level*														
1 Gender	0.52	0.50		—										
2 Age	2.18	0.44		-.01	—									
3 Team tenure	24.55	25.68		.15^†^	.50**	—								
4 LMX	5.30	0.95		.01	-.08	-.03	**(.87)**							
5 Team identification	5.49	0.96		.14^†^	-.06	-.12	.50**	**(.78)**						
6 Colleague support	3.90	0.72		-.04	-.15^†^	-.12	.46**	.26**	**(.81)**					
7 Felt obligation	4.32	0.68		.05	-.05	.01	.65**	.54**	.45**	**(.91)**				
8 Promotive voice	4.79	1.19		.13	-.02	.01	.19*	.09	.13^†^	.30**	**(.93)**			
*Team level*														
9 Team size	8.18		4.76									—	.08	.05
10 Procedural justice climate	5.09		0.70										**(.88)**	-.36**
11 Subgroup formation	2.92		1.09											**(.88)**

*Note*. *N* = 162 for individual level variables, and *N* = 51 for team level variables. Gender was coded as 1 for male and 2 for female. Internal consistency coefficients, Cronbach’s alphas are reported in the parentheses on the diagonal. Individual level correlations are below the diagonal, and team level correlations are above the diagonal.

^†^ *p* < .1,

* *p* < .05,

** *p* < .01.

### Hypotheses testing

**Fig 2:**
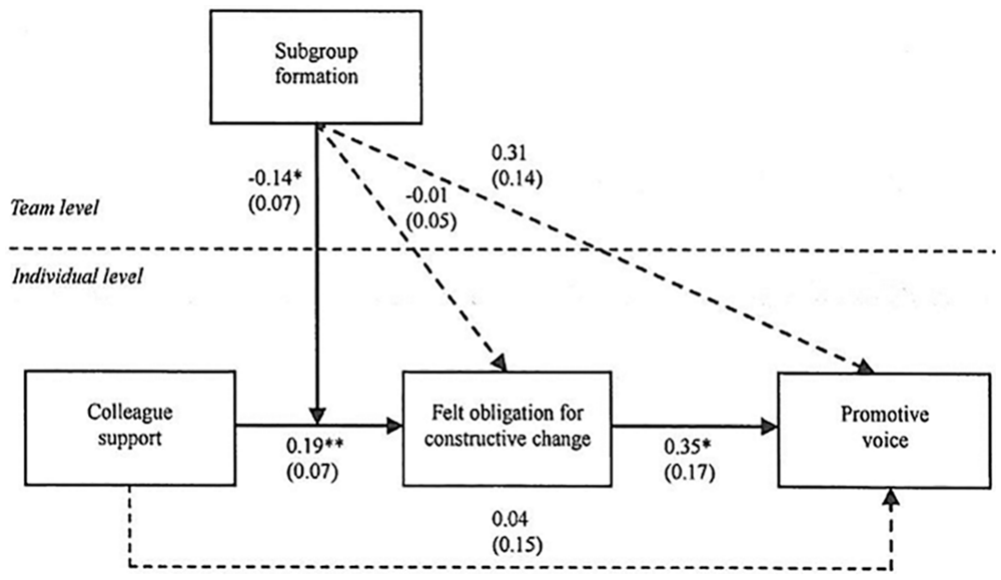
Path coefficients from the selected model. For reasons of brevity, we do not present the effects of team size and procedural justice climate on subgroup formation and individual-level variables or the effects of individual-level gender, age, team tenure, LMX and team identification on felt obligation for constructive change and promotive voice. Interested readers may contact the corresponding author for estimates of these effects. ** *p* < .01, * *p* < .05.

To test the hypothesized mediating effect (i.e., Hypothesis 1) in the hypothesized model (Fig 1), we established a multilevel model that specifies the Level 1 random slope effect of colleague support on felt obligation for constructive change and the Level 1 random slope effects of felt obligation for constructive change on employee promotive voice. The unstandardized coefficient estimates of the hypothesized model are presented in Fig 2. Following Bauer, Preacher, and Gil [53], the covariances among the random slope effects were also estimated to test the Level 1 indirect effects. We controlled for the direct effect of colleague support on the dependent variables. We included gender, age, tenure, LMX and team identification as control variables with fixed effects on employees’ felt obligation for constructive change and promotive voice. We also controlled for the effect of team size and procedural justice climate on team-level subgroup formation and on individual-level employees’ felt obligation for constructive change and promotive voice. To facilitate the interpretation of the research model, individual-level variables, i.e., gender, age, tenure, LMX, team identification and colleague support, were all group mean centered, while team-level variables, i.e., team size, procedural justice climate and subgroup formation, were grand mean centered.

Table 3 presents the unstandardized coefficient estimates for the hypothesized model. With the exception of the effects of LMX on employees’ felt obligation (*γ* = .28, *p* < .01), team identification on employees’ felt obligation (*γ* = .24, *p* < .01), and procedural justice climate on promotive voice (*γ* = .38, *p* < .05), most of the control variables’ effects were not significant. We used Snijders and Bosker’s [54] formulas to calculate the pseudo-*R* ^2^ (~*R* ^2^), which reflects the proportional reduction of individual-level and team-level errors due to the inclusion of predictors in the model [55]. The direct effect of colleague support on promotive voice was not significant. Predictors included in the model accounted for 31.9% and 7.0% of the individual-level variance in felt obligation for constructive change and promotive voice, respectively. These results suggest that individual-level variables, such as colleague support and felt obligation for constructive change, significantly predicted employees’ promotive voice.

#### Hypothesis 1

Felt obligation was hypothesized to mediate the relationship between colleague support and promotive voice. In support of this hypothesis, the results shown in Table 2 suggest that colleague support was positively related to felt obligation for constructive change (*γ* = .19, *p* < .01), and felt obligation for constructive change was also positively related to promotive voice (*γ* = .35, *p* < .05). To estimate the hypothesized indirect relationship, we used a parametric bootstrap procedure [52]. With 20,000 Monte Carlo replications, the results showed a positive indirect relationship between colleague support and employee promotive voice via felt obligation (indirect effect = .067, 95% bias-corrected bootstrap CI [.002, .163]). Therefore, Hypothesis 1 was well supported.

**Table 3:** Unstandardized coefficients of the multilevel model.

	Felt obligation		Promotive voice	
Variables	*Estimate*	*SE*	*Estimate*	*SE*
*Fixed effects*				
Intercept	4.32**	0.05	4.79**	0.13
Gender	-0.05	0.14	-0.10	0.17
Age	-0.10	0.11	-0.14	0.14
Tenure	0.01	0.01	0.01	0.01
LMX	0.28**	0.08	-0.07	0.14
Team identification	0.24**	0.09	0.14	0.12
Team size	0.01	0.01	-0.01	0.02
Procedural justice climate	0.31**	0.07	0.38*	0.18
Colleague support	0.19**	0.08	0.04	0.15
Colleague support * subgroup formation	-0.14*	0.07		
Felt obligation			0.35*	0.17
*Variance components*				
Colleague support slope	0.21**	0.03		
Felt obligation slope			0.61**	0.11
Residual	0.04*	0.03	0.64**	0.19

*Note*.

* *p* < .05,

** *p* < .01.

#### Hypothesis 2

**Fig 3:**
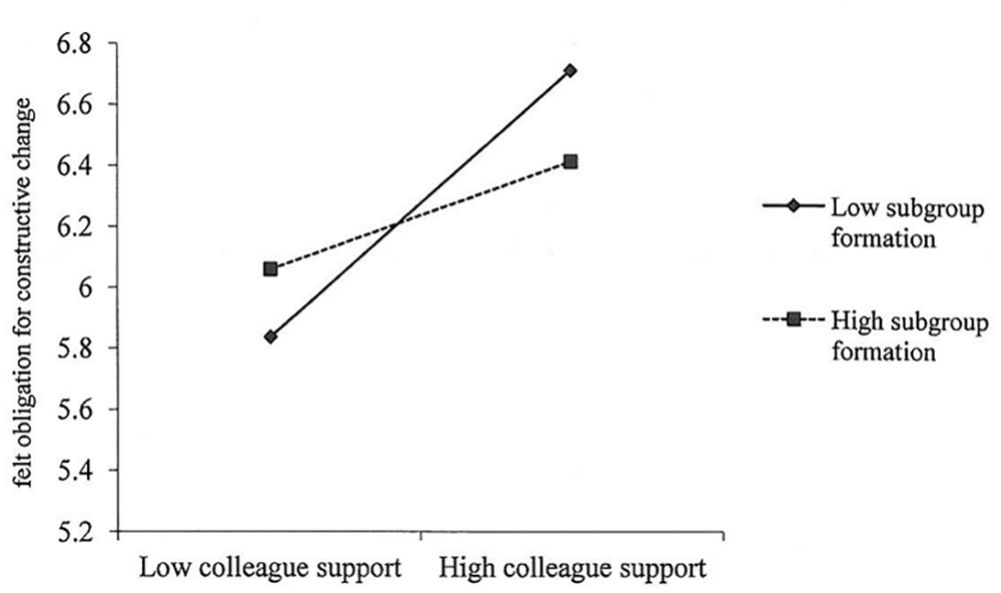
Moderating effect. Subgroup formation moderates the relationship between colleague support and felt obligation for constructive change.

Subgroup formation was hypothesized to moderate the effect of colleague support on felt obligation for constructive change. Fig 2 shows that subgroup formation significantly moderates the relationship between colleague support and felt obligation for constructive change (*γ* = -.13, *SE* = .07, *p* < .05). Following Cohen and colleagues’ [56] recommendations, we plotted this interaction as conditional values of subgroup formation (one standard deviation above and below the mean) in Fig 3. A simple slopes test indicated that colleague support was positively related to felt obligation for constructive change at lower levels of subgroup formation (*γ* = .38, *p* < .01), while this relationship was not significant at higher levels of subgroup formation (*γ* = .05, *p* = .68). Therefore, hypothesis 2 was supported.

#### Hypothesis 3

We further hypothesized that subgroup formation would significantly moderate the effect of colleague support on felt obligation for constructive change, which induces promotive voice. Fig 2 and Table 3 show that felt obligation for constructive change was positively related to promotive voice (*γ* = .35, *p* < .05) and that subgroup formation significantly moderates the relationship between colleague support and felt obligation for constructive change (*γ* = -.14, *p* < .05). A parametric bootstrap procedure (c.f. [52]) was used to estimate the hypothesized cross-level indirect relationship. With 20,000 Monte Carlo replications, the results showed that subgroup formation significantly moderates the relationship between colleague support and employee’s felt obligation for constructive change, which induces promotive voice (indirect effect = -.046, 95% bias-corrected bootstrap CI [-.124, -.001]). These findings support Hypothesis 3.

## Discussion

Using social exchange theory as a basis, the present study aims to (a) explore the impact of felt obligation on employees’ promotive voice behavior; (b) investigate the alternative role of colleague support in predicting employee voice besides personal causes and other contextual factors such as leadership behavior or team climate; and (c) identify the boundary condition for the impact of colleague support using a cross-level framework. Results indicated that colleague support can positively lead to promotive voice by enhancing a sense of obligation to constructively contribute to focal team/organization. Moreover, the relationship between colleague support and employees’ felt obligation for constructive change is salient only when there is a lower degree of team-level subgroup formation.

### Theoretical implications

Our research has several theoretical implications for the employee voice literature. First, we advanced knowledge of antecedents of promotive voice by highlighting the role of felt obligation for constructive changes in enhancing employees’ promotive voice behavior. The majority of current literature emphasized the role of perceived psychological safety in motivating employees to speak up [16, 17]. However, researchers stated that feelings of safety is not enough for encouraging employees to make promotive voice because such promotive voice behavior requires high commitment and continuous effort [1]. Accordingly, we moved beyond such deficiency by exploring felt obligation for constructive change as the mechanism to employee promotive voice.

Second, through the lens of social exchange, we examined the alternative impact of peer colleagues on promotive voice behavior in team settings. Previous voice research indicated that employee voice is primarily predicted by personal causes (e.g., [8]), leader behavior (e.g., [10]) and team climate (e.g., [9, 33]). Less was known regarding the impact of peer colleagues [17, 23]. For an exception, Liu and colleagues [57] found that peer colleagues’ mood may influence employee’s perception of psychological safety to voice. In this study, we demonstrated that colleague support may act as another important source which leads to employee voice. Therefore, this study significantly contributes to the voice literature by demonstrating the impact of colleagues in employee voice processes.

Third, using a cross-level framework, we integrated research on employee promotive voice and subgroup formation in the present study. Answering prior calls for understanding the influence of division among team members (the formation of subgroups) on team dynamics from a multilevel perspective [16], this study demonstrated a salient moderating effect of team level subgroup formation on the relationship between colleague support and felt obligation for constructive change, which subsequently leads to promotive voice.

### Managerial implications

This research is informative for practitioners for several reasons. First, given the nature and potential benefits of promotive voice, it is essential to understand the reasons why employees are willing to speak up [58]. In addition to the influence of leaders, our findings suggest that interpersonal care and support from peer colleagues also can significantly stimulate employees’ promotive voice behavior. As a result, it is strongly recommended that managers should create a supportive climate in which employees receive support and encouragement from the leader and peer colleagues as well.

Second, our findings indicate that employees who have a strong sense of responsibility for the success of the organization are more likely to be motivated to speak up [1]. As a result, it is encouraged that managers should make an effort to improve employees’ understanding of their obligations and responsibilities through frequent communication and training sessions.

Third, subgroup formation may significantly influence the relationship between colleague support and felt obligation for constructive change. Employees who receive support from colleagues will develop a strong sense of obligation for constructive voice only when they are working in a behaviorally and psychologically integrated team. Accordingly, it is critical for managers to use appropriate political skills to create and maintain a cohesive team in which team members are behaviorally and psychologically well integrated. Training and team building sessions on developing a cohesive team are recommended to increase the cross-understanding among team members.

### Strengths, limitations, and future research directions

This research has a number of strengths. First, whereas previous studies have generally focused on the influence of leadership behavior on employee promotive voice, we examine the impact of peer colleagues (i.e., individual level colleague support, team level subgroup formation) on employees’ promotive voice behavior using a cross-level model that controls for the effect of LMX. Second, data were collected from multiple sources, which reduces potential common method biases [59]. Third, the hypothesized model was estimated following a path analytic framework, i.e., all hypothesized relationships were estimated simultaneously. As a result, the problems resulting from piecemeal and causal step approaches to testing mediation [52, 53, 60] were significantly alleviated in the present study. A rigorous empirical examination of the hypothesized multilevel model was provided.

Despite these strengths, several limitations to this study remain. First, in this study we mainly focus on the impact of felt obligation for constructive change on employees’ promotive voice behavior. However, colleague support may also be influential for enhancing employees’ prohibitive voice via other paths [1]. According to Liang and colleagues [1], prohibitive voice is more likely to be expected when employees perceive a high level of psychological safety. Thus, future research is encouraged to explore the effects and mechanisms of colleague support on prohibitive voice. Second, we included LMX in present study to rule out the alternative explanation of the impact of leadership. However, we see the value of taking other relevant factors, such as employees’ proactive personality and psychological safety into consideration to learn more about the reasons why employees voice. Third, there is a limitation regarding statistical conclusion validity because a relatively small-size sample was used to test our hypotheses. It is valuable for future research to verify our empirical results with a larger sample. The final weakness of this study is that data collected from employees and supervisors were measured simultaneously. Although we were not able to measure these variables at different times due to logistical constraints, multi-source data were used to eliminate potential common source bias [59]. Nevertheless, it would be valuable for future research to verify our empirical results with a more rigorous longitudinal design. For example, employees’ promotive voice can be rated by supervisors with a certain time delay. Moreover, employees may report their perceptions of colleague support and felt obligation at two time points with a certain time delay.

## Conclusions

Despite the limitations described above, our study is informative in that it uses the lens of social exchange to investigate how colleague support relates to employee promotive voice by introducing a mediating role of felt obligation for constructive change. We also provided insight as to when these relations are especially likely to manifest, namely, when employees work as a behaviorally and psychologically integrated team. Given that promotive voice has great potential to affect team/organizational outcomes, it is particularly important to understand the cross-level influence mechanisms of employee voice.

## Supporting Information

S1 Data Final full model data. (XLSX)
